# PREDICT: model for prediction of survival in localized prostate cancer

**DOI:** 10.1007/s00345-015-1691-4

**Published:** 2015-09-29

**Authors:** Linda G. W. Kerkmeijer, Evelyn M. Monninkhof, Inge M. van Oort, Henk G. van der Poel, Gert de Meerleer, Marco van Vulpen

**Affiliations:** Department of Radiation Oncology, University Medical Center Utrecht, Heidelberglaan 100, 3584 CX Utrecht, The Netherlands; Julius Center for Health Sciences and Primary Care, University Medical Center Utrecht, Utrecht, The Netherlands; Department of Urology, Radboud University Medical Center, Nijmegen, The Netherlands; Department of Urology, Antoni van Leeuwenhoek Hospital, Netherlands Cancer Institute, Amsterdam, The Netherlands; Department of Radiation Oncology, Ghent University Hospital, Ghent, Belgium

**Keywords:** Grade, Prediction, Prostate cancer, PSA, Survival, T-stage

## Abstract

**Purpose:**

Current models for prediction of prostate cancer-specific survival do not incorporate all present-day interventions. In the present study, a pre-treatment prediction model for patients with localized prostate cancer was developed.

**Methods:**

From 1989 to 2008, 3383 patients were treated with I-125 brachytherapy (*n* = 1694), external beam radiotherapy (≥74 Gy, *n* = 336) or radical prostatectomy (*n* = 1353). Pre-treatment parameters (clinical T-stage, biopsy grade, PSA and age) were related to the hazard of mortality by multivariate Cox proportional hazard analysis. The PRetreatment Estimation of the risk of Death In Cancer of the prosTate (PREDICT) model was developed. The predictive accuracy of the model was assessed by calibration and discrimination and compared to the Ash risk classification system.

**Results:**

Of the 3383 patients analyzed, 2755 patients (81 %) were alive at the end of follow-up, 149 patients (4 %) died of prostate cancer and 365 patients (11 %) died of other causes, and for 114 patients (3 %) cause of death was unknown. Median follow-up time was 7.6 years. After correction for overoptimism, the *c*-statistic of the prediction model for prostate cancer-specific mortality was 0.78 (95 % CI 0.74–0.82), compared to 0.78 (95 % CI 0.75–0.81) for the risk classification system by Ash et al. The PREDICT model showed better calibration than the Ash risk classification system.

**Conclusions:**

The PREDICT model showed a good predictive accuracy and reliability. The PREDICT model might be a promising tool for physicians to predict disease-specific survival prior to any generally accepted intervention in patients with localized prostate cancer.

## Introduction

Many models have been developed for risk stratification of patients with localized prostate cancer. Most nomograms incorporate pre-treatment PSA, clinical T-stage and biopsy Gleason score and are developed to predict biochemical failure for patients undergoing radical prostatectomy (RP), based on either pre-treatment clinical parameters [1–3] or post-treatment clinical parameters [4]. Other nomograms were developed for pre-treatment prediction of biochemical recurrence after external beam radiotherapy (EBRT) [5] or brachytherapy [6]. In some studies, prediction was based on patients treated with either RP or radiotherapy [7]. Although prediction of biochemical failure is important, survival outcome after biochemical recurrence is highly heterogeneous. Since disease-specific survival (DSS) is a more representative measure for significant disease outcome, currently available biochemical risk nomograms are subject of discussion. Generally accepted present-day interventions for localized prostate cancer include I-125 brachytherapy, RP or high-dose (≥74 Gy) EBRT [8–10]. Predictive models that have focused on survival as an outcome measure do not include all present-day standard treatment modalities [11–13]. Other models not included generally accepted current treatment options exclusively [14, 15] and may therefore underestimate survival outcome after treatment in case less effective treatment modalities are included in the model design. In daily practice, due to its general applicability and easy use, risk categories (low, intermediate and high risk) instead of models are frequently used for determining the preferred treatment, for instance the scoring system of Ash and the D’Amico risk classification system, both developed for prediction of biochemical recurrence [16, 17]. The risk category systems, however, do not allow prediction of individual patient survival outcome accurately [18]. In the present study, we aimed to develop a multivariate simple model for prediction of DSS and overall survival (OS) for localized prostate cancer patients prior to treatment with standard present-day interventions (RP, I-125 brachytherapy or high-dose EBRT). The following pre-treatment parameters were taken into account: clinical T-stage, initial PSA (iPSA), biopsy tumor grade and patient age.

## Patients and methods

### Patients and follow-up

Clinical data were collected prospectively for patients treated in four university hospitals in The Netherlands and Belgium. Only patients treated up till 2008 were included in the present study, since sufficient follow-up was required to determine the 10-year probability of DSS and OS. From 1989 to 2008, 1694 patients were treated at the UMC Utrecht Radiotherapy department with Iodine-125 brachytherapy (145 Gy). Implantations were performed according to the GEC-ESTRO guidelines [16]. In the years 1991 up till 2008, 1353 patients were treated by prostatectomy at the Urology Department of the Radboud University Medical Center and the Netherlands Cancer Institute. The method of RP varied (open or laparoscopic or robot-assisted surgery and nerve sparing or non-nerve sparing). Among these 1353 patients, 335 (25 %) have been treated by adjuvant radiotherapy. Between 1998 and 2008, 336 patients were treated at the Ghent University Hospital using an intensity-modulated radiotherapy technique (IMRT) to a dose of 75–78 Gy.

Follow-up was measured until death or the latest known date of being alive (censor date). For the Dutch patients, survival status was retrieved through linkage with the Dutch Cancer Registry at the 31st of December 2012. For the Belgian patients, survival data were derived from the hospitals follow-up program and information from general physicians up till July 2013. Cause of death was retrieved from the medical charts.

### Pre-treatment predictors

Clinical data were derived from the patient’s medical records. Predictive clinical parameters were pre-specified based on the literature to prevent overfitting. Pretreatment T-stage, grade (throughout time different Gleason score/tumor grade definitions have been used), initial PSA (iPSA) and age were incorporated in the PREDICT model. The American Joint Committee TNM classifications were used for determination of the T-stage [19]. To compare the predictive accuracy of the prognostic model with standard risk classification systems, patients were also classified by the Ash classification system (low-risk: T1c-T2a, PSA < 10 ng/mL, Gleason score ≤ 6; intermediate-risk: T2b-T2c, PSA 10–20 ng/mL, Gleason score 7; high-risk: ≥T3a, PSA > 20 ng/mL, Gleason score ≥ 8 and/or ≥2 intermediate-risk factors) [16].

### Statistics

Kaplan–Meier analysis was used to estimate the actuarial DSS and OS. Baseline clinical characteristics and treatment modality are listed in Table 1. For 263 patients (7 %), one or more clinical parameters could not be retrieved (missing completely at random). Imputed missing clinical parameters were used by single imputation to prevent bias that would have occurred if only complete cases were used in the analysis [20]. When compared to the complete case analyses (Table 1), measures of central tendency and dispersion did not change significantly after imputation. Survival outcome was not imputed. The potential predictive parameters including T-stage (T1, T2 and ≥T3a), grade (1, 2 and 3), iPSA (log PSA) and age (age at treatment per 5 years) were related to the hazard of mortality by means of multivariate Cox regression. Cox regression analysis was used for its ability to use censored observations. The values for iPSA and age automatically centered around the mean in the statistical analysis (therefore a negative number of points can be obtained). The ability of the model to discriminate between 10-year mortality or survival was calculated by means of *c*-statistics (Harrell’s concordance index). Since a model’s predictive accuracy is generally too optimistic, internal validation was performed by bootstrapping (*n* = 200) to correct for this overfitting [21]. When using bootstrapping techniques, a model’s performance better reflects the expected performance in a new population of patients. By means of shrinkage of the β-coefficients, the model was adjusted for this overoptimism. The final model was transformed into a clinical prediction rule based on the β-coefficients after shrinkage. The predictive accuracy of the final PREDICT model was evaluated by calibration (reliability) and discrimination. Calibration was assessed by plotting the deciles of the predicted risks of survival (median predicted percentage per decile) compared with the observed risks of survival (10-year DSS derived from actuarial life tables Kaplan–Meier analysis). The prognostic performance of the PREDICT model was compared to that of the risk classification system for prostate cancer by Ash et al. [16].

**Table 1:** Clinical baseline characteristics of localized prostate cancer patients treated by brachytherapy, prostatectomy and external beam radiotherapy

	Brachytherapy	Prostatectomy	EBRT	Total
No. of patients	1694	1353	336	3383
Age (years, mean, range)	66 (43–91)	61 (42–80)	66 (41–80)	64 (41–91)
iPSA (ng/mL, median, range)	8.9 (0.3–100)	8.0 (0.1–157)	10.3 (0.04–302)	8.7 (0.04–302)
Clinical T-stage (median, range)	cT1 (cT1–cT2c)	cT2a (cT1–cT4)	cT2a (cT1–cT4)	cT1 (cT1–cT4)
Biopsy grade (median, range)	1 (1–3)	1 (1–3)	1 (1–3)	1 (1–3)

Descriptive statistics and imputation were performed with the Statistical Package for Social Sciences (SPSS), version 20.0 (Chicago, IL, USA). All other statistical analyses were carried out in the statistical package of R version 2.10 (free software from the GNU project).

## Results

Of the 3383 patients analyzed, 2755 patients (81 %) were alive at time of the censor date (31st of December 2012), 618 patients (18 %) died, and for 10 patients follow-up of survival status could not be retrieved. Among the 618 patients who did not survive, 149 patients (4 %) died of prostate cancer and 365 patients (11 %) died of non-prostate cancer-related death, and for 114 patients (3 %) cause of death was not known. Median follow-up time was 7.6 years (mean 8.3 years, SD 3.8 years), and 958 (28 %) patients reached 10.0-years follow-up (Table 2). According to the Ash risk classification system, 1198 (35 %) patients were categorized as having low-risk prostate cancer, 1151(31 %) as having intermediate-risk disease and 1034 (34 %) as having high-risk cancer.

**Table 2:** Follow-up, disease-specific and overall survival for patients with localized prostate cancer per treatment modality (brachytherapy, prostatectomy and external beam radiotherapy)

	Brachytherapy	Prostatectomy	EBRT	Total
No. of patients	1694	1353	336	3383
Follow-up (years, mean, 95 % CI)	7.9 (7.7–8.0)	9.1 (8.8–9.3)	7.1 (6.8–7.4)	8.3 (8.1–8.4)
10 year DSS (95 % CI)	91 % (90–92)	96 % (95–97)	93 % (90–95)	94 % (93–95)
10 year OS (95 % CI)	72 % (70–74)	87 % (85–89)	77 % (72–81)	79 % (78–80)

*DSS* disease-specific survival, *OS* overall survival

**Fig. 1:**
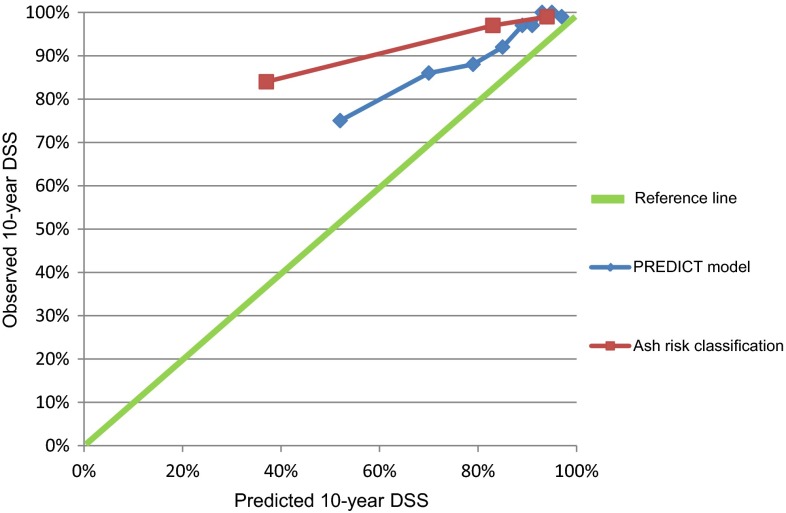
Calibration plots for prediction of disease-specific survival for the PREDICT model versus the Ash risk classification system. The predicted probability of survival based on the PREDICT model was compared to the observed survival. On the *x*-axis the predicted probability and on the *y*-axis the observed (actuarial life table) probability of 10-year disease-specific survival is shown. A slope of ‘1’ (*reference line*) is associated with optimal calibration

Multivariate Cox regression analyses showed a *c*-statistic (before bootstrapping) of the PREDICT model for prediction of DSS of 0.81 (SD 0.03) and 0.68 (SD 0.02) for prediction of OS. In comparison, the corresponding *c*-statistics were 0.78 (SD 0.03) for DSS and 0.61 (SD 0.02) for OS for the risk classification system by Ash et al. The model was corrected for overoptimism (4 % for DSS and 2 % for OS). For the Ash risk scoring system, 0 versus 1 % overoptimism was found. The adjusted β-coefficients for the PREDICT model for prediction of DSS after shrinkage are shown in Table 3. The β-coefficients were rounded (multiplied by 10) to obtain a practical prediction rule. The *c*-statistics of the final PREDICT model after bootstrapping was 0.78 (95 % CI 0.74–0.82) for disease-specific survival. In comparison, a *c*-statistics of 0.78 (95 % CI 0.75–0.81) was calculated for the Ash risk score. For prediction of overall survival, a *c*-statistic of 0.68 (95 % CI 0.65–0.70) was found for the PREDICT model, compared to 0.61 (95 % CI 0.58–0.63) for the scoring system by Ash et al. Figure 1 shows the calibration of the predicted versus the observed risk of disease-specific survival (compared in risk strata) for the PREDICT model versus the Ash risk scoring system.

**Table 3:** Multivariable predictors (after shrinkage) of disease-specific survival for prostate cancer for the PREDICT model

Predictor	HR	95 % CI
Age		
Age per 5 years	1.2	1.1–1.4
Pretreatment T-stage		
cT1 (reference)		
cT2	1.7	1.2–2.5
≥cT3a	1.8	0.9–3.6
Pretreatment grade		
G1 (reference)		
G2	2.9	2.0–4.2
G3	3.6	2.1–6.1
Pretreatment PSA		
Log PSA	4.1	2.6–6.4

The total risk score of the PREDICT model for predicting disease-specific survival is calculated by summing up the number of points obtained for each clinical factor (age + T-stage + grade + iPSA = total score; Table 4). In this table, also the corresponding predicted 10-year disease-specific survival rate can be found for different calculated total risk score categories.

**Table 4:** Pre-treatment tool to predict the 10-year disease-specific survival for patients with localized prostate cancer (PREDICT model)

Age (years)^a^															
Value	44	49	54	59	64	69	74		79	84		89			
Score	−8	−6	−4	−2	0	2	4		6	8		10			
T-stage															
Value					cT1				≥cT2						
Score					0				6						
Grade															
Value					G1								G2	G3	
Score					0								11	13	
iPSA (ng/mL)^a^															
Value		2	3	6	9	13	16	20	25	32	38		50	79	
Score		−6	−4	−2	0	2	4	5	6	8	9		11	13	
															=
Total sum score															
Total sum score	<3		3–7		8–11		12–16		>16						
10-year DSS (%)	≥93		88–92		82–87		72–81		≤71						

^a^Choose the closest value when the patient’s value lies between the given values

## Discussion

After diagnosis of prostate cancer, patients are typically classified into risk categories bases on T-stage, Gleason score and pre-treatment PSA level, most frequently by using the Ash or D’Amico risk classification system [16, 17].

The PREDICT model was developed as an easy tool for use in clinical practice. The goal was to achieve a more reliable prediction of disease-specific survival outcome for individual patients than the existing three category risk classification systems. Both the PREDICT model and the Ash system have a good accuracy for pre-treatment prediction of DSS in non-metastasized prostate cancer (*c*-statistic 0.78). The PREDICT model, however, had a better calibration performance (reliability) than the Ash risk classification system. In other words, the predicted 10-year DSS is closer to the observed 10-year Kaplan–Meier derived DSS for the PREDICT model than for the Ash risk classification.

Since the model was developed on patients treated by well-established present-day types of treatment only (brachytherapy, high-dose EBRT, RP), this model will be applicable to the majority of prostate cancer patients. Accurate prediction of treatment outcome is essential for patient counseling. The PREDICT model was compared to the Ash risk classification, since this risk classification is frequently used in clinical practice to decide on the treatment modality of preference. Overall survival could not be accurately predicted by the PREDICT model nor by the Ash risk classification. This can be explained by the fact that prostate cancer mortality is only a small proportion of overall mortality, and only prostate cancer-related factors (not comorbidity parameters, except for patient age) were incorporated in the present model design. Walz et al. [22] did develop a model specifically for predicting 10-year general life expectancy with a high predictive accuracy (84 %) including age and Charlson comorbidity score. Other models have been used to predict life expectancy for non-prostate cancer-related causes [23].

As we expected, our predictive model allows for better individual prediction of DSS than the Ash risk score system. Risk classification relies on extrapolation of several risk factors in a heterogeneous patient group [18]. The strengths of the PREDICT model are the large patient numbers included. The median follow-up is 7.6 years (mean 8.3 years). Furthermore, the whole spectrum of current interventions and only patients treated by well-established interventions (with assumed comparable cure rates) for prostate cancer were incorporated. The low-, intermediate- and high-risk patient groups according to the Ash risk score system were equally represented in the database. A possible limitation of the present study is that for 3 % of patients cause of death could not be retrieved (equally divided among RP and brachytherapy patients, one missing cause of death among EBRT patients). Furthermore, patients were treated in different centers, with possible differences in pre-treatment imaging, number and system of taking biopsies and pathology evaluation (inter-observer variability and Gleason score and tumor grade definition throughout time). Huang et al. [24] proposed integration of percentage of positive cores in risk stratification models. However, the percentage of positive cores was not available for a large number of patients in our database and could therefore not be used. The effect of treatment modality (brachytherapy, RP or high-dose radiotherapy) on DSS is not known, since no randomized controlled trials are available up to date. The evidence available is conflicting and was based on retrospective data [25, 26]. In the present study, we have assumed that DSS is comparable in all three treatment modalities and only dependent on risk factors. T-stage was the weakest predictor in the PREDICT model. T-stage and also tumor grading are known to be subject to understanding by random (and extended) biopsies and digital rectal examination/transrectal ultrasound when compared to post-prostatectomy pathology results [27, 28]. Adding MRI-based T-staging may increase the predictive accuracy of a predictive model. Prediction of biochemical recurrence after EBRT indeed improved by MRI imaging in addition to the Kattan model, when compared to the Kattan model alone [29].

Originally, pre-treatment risk category systems and models were developed to predict biochemical disease control. The Kattan, Stephenson and CAPRA models are the ones mostly used and have been extensively externally validated [1–3, 30]. A significant proportion of patients with biochemical recurrence, however, does not die from prostate cancer; therefore, endpoints for unambiguous disease outcome such as survival or risk of metastatic disease are more relevant, but require longer follow-up time. Although many predictive models for prostate cancer exist, a minority of models is developed to predict survival and there is a lack of models for survival prediction incorporating all generally accepted present-day interventions [11–15]. Two CAPRA (Cancer of the Prostate Risk Assessment) score-based models have been developed. The model design, however, did not exclusively include patients treated by current interventions [14, 15]. Particularly, EBRT dose and technique were heterogeneous in both studies. Radiation therapy patients were included from 1987 on, and a lower dose to the prostate has been associated with an inferior tumor control and a higher risk of metastatic spread [10]. Indeed, the model showed a lower discriminative capacity for prediction of survival in patients treated by radiation compared to the prostatectomy group. In the study of Cooperberg et al. [15] also, patients managed by watchful waiting, active surveillance, primary hormonal therapy and cryotherapy were included, therefore biasing survival outcome to a lower survival rate when using the model for patients treated by more effective modalities. For the Kutikov et al. [14] model, discriminative capability of the model was not described, which makes it hard to evaluate the internal validity of the model. Both CAPRA-based models were not externally validated for prediction of prostate cancer-specific mortality. When developing a model, several important considerations should be taken into account [18]. External validation is the golden standard for evaluating reproducibility and accuracy. The PREDICT model was based on large patient numbers with an intermediate follow-up time, has a good discrimination and calibration, but has to be validated in an external dataset before its clinical application. For (internal) validation purposes [21], we did perform bootstrapping in anticipation of the external validation study, which will be performed on a different dataset.

## Conclusion

Available pre-treatment models developed for prediction of survival after treatment for prostate cancer either not include all present-day interventions or include suboptimal treatment options or active surveillance. After external validation, the PREDICT model might be a promising tool for physicians to predict survival preceding any current generally accepted intervention for non-metastasized prostate cancer.
